# Supplementary material for: Lumpy Skin Disease Virus ORF137 Protein Inhibits Type I Interferon Production by Interacting with and Decreasing the Phosphorylation of IRF3
Source: Cells. 2025 Sep 22;14(18):1475. doi: 10.3390/cells14181475 (PMC12468088; doi:10.3390/cells14181475)

Supplementary information

Figure S1. The detection of interaction between LSDV ORF137 with cGAS, STING and TBK-1. **(A, C, E-F)** 293T cells were transfected with plasmids hs-cGAS-HA (4 μg), Bos-cGAS-Flag (4 μg), hs-STING-HA (4 μg), Bos-STING-Flag (4 μg), hs-TBK1-HA (4 μg) and Bos-TBK1-HA (4 μg) respectively together with control vector pCAGGS-Flag (4μg) or ORF137-Flag (4 μg) 24 h later, cells were collected and Co-IP assay were used to detect the interaction of ORF137 with these proteins. **(B, D)** 293T cells were transfected with plasmids Bos-cGAS-Flag (4 μg) and Bos-STING-Flag (4 μg) respectively together with control vector pCMV-HA (4 μg) or ORF137-HA (4 μg) 24 h later, cells were collected and Co-IP assay were used to detect the interaction of ORF137 with these proteins.

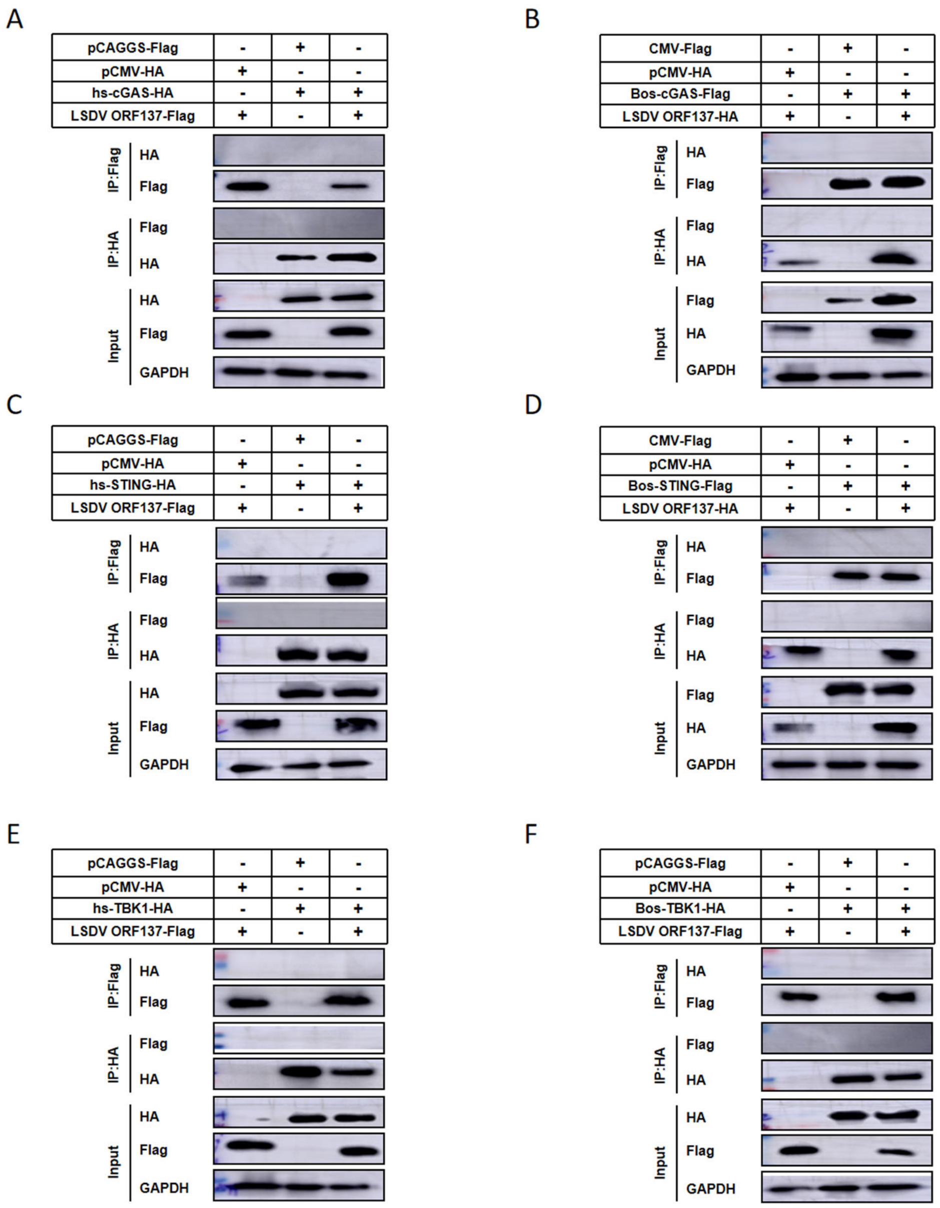

Supplement: Supplementary file 1 [file cells-14-01475-s001.zip › cells-3894858-supplementary.pdf]
